# Balancing health benefits and social sacrifices: A qualitative study of how screening-detected celiac disease impacts adolescents' quality of life

**DOI:** 10.1186/1471-2431-11-32

**Published:** 2011-05-10

**Authors:** Anna Rosén, Anneli Ivarsson, Katrina Nordyke, Eva Karlsson, Annelie Carlsson, Lars Danielsson, Lotta Högberg, Maria Emmelin

**Affiliations:** 1 Department of Public Health and Clinical Medicine, Epidemiology and Global Health, Umeå University, Umeå, Sweden; 2 Department of Medical Biosciences, Medical and Clinical Genetics, Umeå University, Umeå, Sweden; 3 Pediatrics, Växjö Hospital, Växjö, Sweden; 4 Department of Clinical Sciences, Pediatrics, Lund University, Lund, Sweden; 5 Pediatrics, Norrtälje Hospital, Norrtälje, Sweden; 6 Department of Clinical and Experimental Medicine, Pediatrics, Linköping University, Linköping, Sweden; 7 Department of Clinical Sciences, Social Medicine and Global Health, Lund University, Lund, Sweden

## Abstract

**Background:**

Celiac disease often goes undiagnosed. Mass screening might be an option to reduce the public health burden of untreated celiac disease. However, mass screening is still controversial since it is uncertain whether the benefits of early detection outweigh the possible negative consequences. Before implementation of screening programs, the experiences of those being identified as cases should be considered. The aim of our study was to explore how screening-detected celiac disease impacts adolescents' quality of life, as perceived by themselves and their parents.

**Methods:**

All adolescents (n = 145) with screening-detected celiac disease found in a Swedish screening study, and their parents, were invited to share their experiences in a qualitative follow-up study. In total, we have information on 117 (81%) of the adolescents, either from the adolescents themselves (n = 101) and/or from their parent/s (n = 125). Written narratives were submitted by 91 adolescents and 105 parents. In addition, 14 focus group discussions involving 31 adolescents and 43 parents were conducted. Data was transcribed verbatim and analyzed based on a Grounded Theory framework.

**Results:**

The screening-detected celiac disease diagnosis had varying impact on quality of life that related both to changes in perceived health and to the adolescents' experiences of living with celiac disease in terms of social sacrifices. Changes in perceived health varied from *"healthy as anyone else with no positive change" *to *"something was wrong and then changed to the better"*, whereas experiences of living with celiac disease ranged from *"not a big deal" *to *"treatment not worth the price"*. Perceptions about living with celiac disease and related coping strategies were influenced by contextual factors, such as perceived support from significant others and availability of gluten-free products, and were developed without a direct relation to experiencing changes in perceived health.

**Conclusions:**

Screening-detected celiac disease has varying impact on adolescents' quality of life, where their perceived change in health has to be balanced against the social sacrifices the diagnosis may cause. This needs to be taken into account in any future suggestion of celiac disease mass screening and in the management of these patients.

## Background

Celiac disease (CD), also called gluten intolerance, is a chronic autoimmune enteropathy triggered by ingestion of gluten [1]. Clinical manifestations range from minimal to severe and can include tiredness, stomach ache, diarrhea or constipation, weight loss, and anemia [2,3]. CD is also associated with an increased risk of long-term negative health consequences such as short stature, delayed puberty, depression, and low bone mineral density [1]. Serological markers facilitate the recognition of CD cases, but definitive diagnosis requires histological alterations of the small bowel mucosa [4]. The only available treatment is a lifelong strict gluten-free diet, i.e. exclusion of all food containing wheat, rye, and barley, which usually restores the mucosa and resolves symptoms [1].

Screening studies of pediatric populations in different parts of the world have revealed a CD prevalence varying between 3/1000 and 56/1000, always with a higher proportion of previously undiagnosed cases [5-9]. Thus, current guidelines focused on active case finding and testing risk groups do not identify the majority of cases. Mass screening might be an option to reduce the public health burden of untreated CD. The World Health Organization's criteria for implementation of mass screening programs are partly fulfilled; CD is a common disease and often unrecognized, suitable serological markers to use in a screening are available, and gluten-free diet is an effective treatment [10,11]. Still, mass screening is controversial [11-18]. Long term follow-up studies of untreated CD cases and health economic evaluations are needed. Moreover, it is uncertain whether early detection by screening outweighs the harm possibly caused when someone, who probably has perceived oneself as relatively healthy, becomes diagnosed with a chronic disease requiring lifelong dietary restrictions.

Previously, screening-detected CD cases were assumed to be asymptomatic, but it is now evident that screening also detects symptomatic cases [19,20]. Treatment with gluten-free diet has been reported to have beneficial effects on symptoms and quality of life of screening-detected CD children [21]. However, to be diagnosed with CD, and adhere to lifelong dietary restrictions, poses challenges which may also have a negative effect on quality of life [22,23].

Further studies exploring how a screening-detected CD diagnosis affects well-being and daily life, especially when diagnosed during childhood and adolescence, are warranted [11,16,18]. Previous studies on CD and quality of life have mainly employed quantitative methods [21-26]. However, to allow for an in-depth exploration of experiences we chose a qualitative approach. The aim was to explore how screening-detected CD impacts adolescents' quality of life, as perceived by themselves and their parents.

## Methods

### Design

An explorative study design with a qualitative research approach was chosen to capture the participants (informants) own descriptions of their major concerns [27-29]. Qualitative research methodology includes systematic collection and interpretation of text derived from written narratives, individual interviews, or focus group discussions [30,31]. Such methods are widely used in health care research to capture experiences, thoughts, attitudes, and processes - all core components of clinical knowledge. In our study, written narratives aimed to capture individual experiences, whereas focus group discussions facilitated interaction between informants to explore group norms, attitudes, and processes. Informed consent was given by all informants and the study had ethical approval from the Regional Ethical Review board in Umeå, Sweden [Dnr UmU 04-156-M].

### The setting

A school based CD screening of 12-year olds in Sweden was conducted in 2005 - 2006 [5]. In total, 10041 adolescents were invited with 75% participating. Blood samples from 7208 children (3467 girls, 3741 boys), without previously known CD, were analyzed for CD serological markers, rendering 145 screening-detected cases (75 girls, 70 boys) verified by biopsy. They were all advised to follow a gluten-free diet.

### Informants

All adolescents with screening-detected CD (n = 145), and their parents, were invited to this follow-up study. We have information on 117 (81%) of the adolescents, either from the adolescents themselves (n = 101) and/or from their parent/s (n = 125). Written narratives were submitted by 91 adolescents and 105 parents. In addition, 14 focus group discussions were held, involving 31 adolescents and 43 parents. Of the adolescents, 70 wrote only narratives, 21 wrote narratives and participated in a focus group discussion, and 10 participated only in a focus group. Out of the parents that participated, 82 wrote only narratives, 23 wrote narratives and participated in a focus group discussion, and 20 participated only in a focus group. These parents represented families of 111 adolescents, i.e. from some families both parents participated. Characteristics of the informants are given in Table 1.

**Table 1:** Characteristics of adolescents with screening-detected celiac disease (CD), and their parents, participating in the study

**Adolescents**	
**Adolescents (n)**	101
Girls	53
Boys	48
**Age in years**^a^	14.6 (13.9-15.4)
**Months since diagnosis**^a^	16.9 (11.1-23.2)
**Basis for CD diagnosis (n)**	
Subtotal/total villous atrophy	61
Partial villous atrophy	27
Borderline mucosa^b^	13
**Parents**	
**Parents/families (n)**	125/111
Mothers	94
Fathers	27
Gender not specified^c^	4
**Education (n)**	
Primary	5
Secondary	60
University degree	46
Education not specified	14

^a ^Median (Range)

^b ^Borderline mucosa, that is >30 intraepithelial lymphocytes (IEL) per 100 enterocytes, in combination with symptoms and/or signs compatible with CD

^c ^Contributed with narratives only

In conjunction with writing narratives, adolescents answered two multiple choice questions concerning: i) self-reported compliance with gluten-free diet (response alternatives were always, often, sometimes, and never) and ii) perceived well-being today compared to before the CD diagnosis (response alternatives were much better, somewhat better, no difference, somewhat worse, and much worse). These questions were responded to by 93 of the 101 participating adolescents. Table 2 describes compliance with gluten-free diet and perceived change in well-being among our sample of adolescents. Reported compliance to the gluten-free diet was as follows: always 72.0%, often 25.8%, sometimes 0%, and never 2.2%. Out of these adolescents, 53.8% perceived that they felt much or somewhat better now compared to before the diagnosis, 36.6% reported no difference, 4.4% that they felt somewhat or much worse, and 5.4% did not remember.

**Table 2:** Compliance with gluten-free diet and change in well-being one year after diagnosis

		Compliance with gluten-free diet					
	Response alternatives	Always gluten-free n	Often gluten-free n	Sometimes gluten-free n	Never gluten-free n	Total n	(%)

**Well-being today compared to before the CD diagnosis**	Much better	22	10	0	0	32	(34.4%)
	Somewhat better	12	6	0	0	18	(19.4%)
	No difference	26	7	0	1	34	(36.6%)
	Somewhat worse	2	0	0	0	2	(2.2%)
	Much worse	0	1	0	1	2	(2.2%)
	Do not remember	5	0	0	0	5	(5.4%)
	
	Total n (%)	67 (72.0%)	24 (25.8%)	0 (0%)	2 (2.2%)	93	

Compliance with gluten-free diet, and well-being today compared to before the CD diagnosis, as reported by the screening-detected celiac disease adolescents participating in the study (93 respondents out of 101 adolescents).

### Written narratives

Adolescents, and their parents, were mailed invitations to write narratives, with instructions encouraging them to individually share their experience of the adolescent's CD diagnosis, and specifically to elaborate on any change in perceived health and other daily life consequences. The length of the narratives ranged between 1-2 handwritten pages, which were all transcribed verbatim.

### Focus group discussions

Adolescents and parents attended separate groups but were mixed in terms of gender. The discussions were moderated by the principal author and one of the co-authors. Hypothetical scenarios and drawings, illustrating various aspects of living with CD and a gluten-free diet, were used to stimulate the discussions. The sessions lasted 55-90 minutes, were digitally recorded, and later transcribed verbatim. Reflective notes were continuously taken to guide the subsequent group discussions.

### Analysis

**Figure 1:**
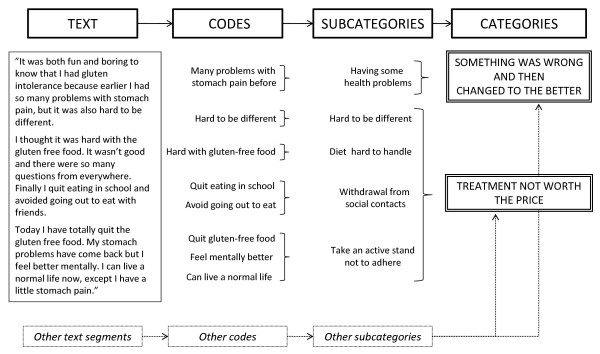
**The process of Grounded Theory analysis, moving from the text to theoretically constructed categories**.

The analysis was based on a Grounded Theory framework [31], with the aim to develop a conceptual model of how the screening-detected CD diagnosis impacted quality of life. The transcribed texts were read several times and entered into Open Code software [32]. The text was subjected to an open coding process to conceptualize information of importance for the research question. The codes were compared for commonalities and clustered as a basis for developing sub-categories. As a final step, the sub-categories were examined to construct the categories for the final model. Sub-categories and categories were continuously compared with the original text to ensure that the results were well grounded in the data. An overview of the analytical process is given in Figure 1.

## Results

**Figure 2:**
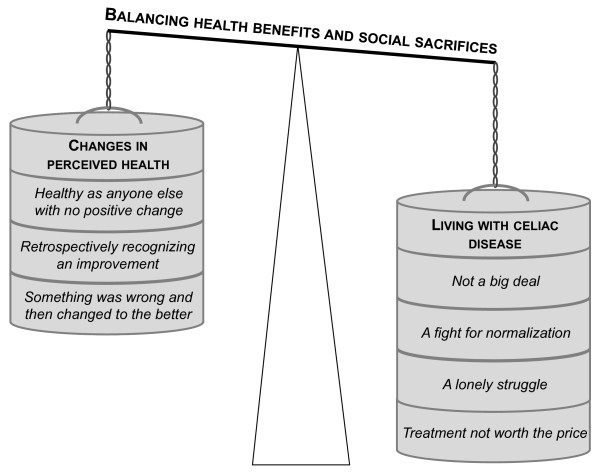
**A conceptual model of how a screening-detected CD diagnosis impacts on adolescents' quality of life**. The model illustrates that changes in perceived health have to be balanced against adolescents' experiences of living with celiac disease. Thus, the impact on quality of life can be characterized as balancing health benefits and social sacrifices.

The impact of a screening-detected CD diagnosis on quality of life can be characterized as balancing health benefits and social sacrifices, as illustrated in the conceptual model in Figure 2. The categories show that changes in perceived health after diagnosis ranged from *"healthy as anyone else with no positive change" *to *"something was wrong and then changed to the better"*. However, since the social consequences of the disease and the treatment were given much attention by the adolescents, the model also includes categories reflecting their experiences of living with CD. These categories ranged from *"not a big deal" *to *"treatment not worth the price" *and had no direct relation to changes in perceived health. Thus, those with great health benefits could be found among those suffering most in terms of social consequences and vice versa.

In the following text we describe more in detail impact on quality of life with respect to changes in perceived health and experiences of living with CD, with accompanying categories presented as subheadings. Quotations from adolescents and parents show how our interpretations are grounded in the data.

### Changes in perceived health

Adolescents and their parents described a wide range of signs and symptoms before diagnosis and as a result the CD diagnosis had varying impact on quality of life in terms of perceived health benefits. Table 3 illustrates how both adolescents' own experiences and parents' observations of signs and symptoms before and after diagnosis relates to the three categories reflecting overall changes in perceived health.

**Table 3:** Categories reflecting overall changes in perceived health

Quotations (Original text)	BEFORE diagnosis and initiated treatment (Subcategories)	AFTER diagnosis and initiated treatment (Subcategories)	Changes in perceived health (Categories)
*"I felt good before and I felt good after, so this wasn't a * *big change for me."* Boy, Focus group discussion	Perfectly healthy (A) Nothing was wrong (A+P) Happy and healthy as anyone else (P)	No positive change (A) No difference in well-being (P) Stomach ache if eating gluten (A)	***Healthy as anyone else with *** ***no positive change***

*"I thought that I was pretty energetic and all, but when I found out about * *it and started eating like this, I've noticed that I have become much * *more energetic than I was before."* Boy, Focus group discussion	Nothing was wrong (A+P) Thought it was personality (P) Did not like bread (A) Avoided food like bread and pasta (P)	Feeling better (A) Improvement in well-being (P) Everything fell into place (A+P)	***Retrospectively recognizing an *** ***improvement***

*"With the CD-diagnosis we got an explanation to her frequent problems * *with stomach ache, vomiting without reasons, long intense infections, * *her tiredness, and that she looked so worn. She is much happier and more energetic now."* Mother to a daughter, Narrative	Having some health problems (A) Several signs of illness (P) Having a different mood (A+P) Something was wrong (P)	Feeling better (A) Improvement in well-being (P) Not that angry anymore (A+P) A NEW child (P)	***Something was wrong and then *** ***changed to the better***

Quotations illustrating the grounding of theoretically constructed subcategories, reflecting understanding of symptoms and perceived health before and after diagnosis, as described by adolescents (A) and their parents (P), which finally are grouped into categories reflecting overall changes in perceived health.

#### Healthy as anyone else with no positive change

This category referred to those perceiving themselves as healthy before diagnosis and who did not notice any positive change after initiated treatment. A few even described that occasional intake of gluten containing food now gave them a stomach ache, a phenomena which they had not experienced before initiated treatment. As the adolescents emphasized that there was nothing wrong with them, a view their parents shared, some were reluctant to believe that the diagnosis was even correct.

"When I found out that I was probably gluten intolerant I thought it was a mistake. I was as healthy as can be."

Boy, Narrative

#### Retrospectively recognizing an improvement

This category captured the experience of those who considered themselves to be asymptomatic before diagnosis, but became aware of symptoms when receiving the diagnosis or when perceiving improved health after initiated treatment. One girl described how she retrospectively became aware of her symptoms and that everything fell into place.

"I had never had clear symptoms; sure I had been kind of tired during the day and didn't want to be with friends after school. When we thought about it later, everything fell into place. It was my diet that had made me so tired [...]. Now I feel much better. I am happier, have more energy and want to do more!"

Girl, Narrative

Interestingly, some parents had reflected on that their child seemed to be different, but did not suspect this was caused by a disease. Instead, they thought the adolescents' behavior was due to teenage problems or associated with the personality. A mother described how she had thought she had an anxious daughter.

"We thought that we had a child who was very worried and nervous because she was always running to the toilet. We didn't connect that this had to do with the food!"

Mother to a daughter, Narrative

Receiving the diagnosis gave an increased understanding of symptoms and retrospective insight about previously unrecognized symptoms. However, parents also stated that they may be prone to try to find health improvement to justify that something good came out of receiving the diagnosis.

#### Something was wrong and then changed to the better

This category reflects improved well-being among those with previous signs of illness. A wide variety of symptoms was mentioned, e.g. a feeling of low energy, tiredness, paleness, dizziness, being angry, having a different mood, not growing well, being thin, mouth blisters, stomach ache, delayed puberty, diarrhea, and vomiting. We noted a pattern where parents described the girls as being tired and without energy, whereas boys were perceived to have a bad overall mood. After initiated treatment, both the adolescents and parents described how they sensed a considerable improvement in the adolescents' health.

"He used to be constantly tired and out of sorts, had a hard time concentrating and easily lost his temper. He also occasionally had slight pains in the stomach and heart area. Now our son is energetic and pleasant, even though he is at the same time entering his teens. Wonderful!"

Mother to a son, Narrative

Parents described trying to find explanations for observed symptoms. Frequent stomach ache had, for example, raised suspicion of the adolescent having difficulties at school or with friends. Some had sought medical help, without the CD being diagnosed. A father described how his daughter's symptoms were blamed on psychosocial issues, although he did not fully believe this himself.

"We had visited the clinic for different things earlier. My daughter had never had stomach pain [...]. What she had was poor levels of iron and joint pain in the fingers. It ended with them explaining that it was psychological and they hinted, in a roundabout way, that it was because she has a handicapped sibling. That's how it ended, so to say."

Father to a daughter, Focus group discussion

### Living with CD

Adolescents' feelings and attitudes towards the CD diagnosis, together with related actions, resulted in different experiences of living with CD, which in turn had varying impact on quality of life. Table 4 shows the links between expressed feelings and attitudes, actions taken, and the four categories constructed to describe the variation in experiences of living with CD, in terms of social sacrifices. These categories can be seen as typologies that are grounded in empirical data. No attempt was made to categorize specific adolescents into the different typologies, since one adolescent can contribute to more than one category and the adolescents may move back and forth between the categories over time.

**Table 4:** Categories reflecting experiences of living with celiac disease

Quotations (Original text)	Feelings and attitudes (Subcategories)	Related actions (Subcategories)	Experiences (Categories)
*"When I found out that I have gluten intolerance, there wasn't anything to it. Of course you want to be able to eat what you want, but if you can't then you just can't."* Girl, Narrative	Not a disease, just food intolerance Not the worst condition to have Not being the only one	Accept the fact Make the best out of it	***Not a big deal***

*"It should be out in the national newspaper, like one day they should have a bunch about gluten. So people understand."* Boy, Focus group discussion	Wish for increased awareness Dislike to be treated differently Want to be seen as normal	Mobilize for change Educate others	***A fight for normalization***

*"My life became very different. They always have to make special food for me and I am scared that people think that I am being difficult."* Girl, Narrative	Feel alone Worry to be seen as a burden Feel like an outsider	Trying to hide Compensate by being nice	***A lonely struggle***

*"I have totally quit the gluten-free food. My stomach problems have come back, but I feel better mentally. I can live a normal life now, except I have a little stomach pain."* Girl, Narrative	Hard to be different Diet hard to handle Not worth it	Withdrawals from social contacts Take an active stand not to adhere	***Treatment not worth the price***

Quotations related to experiences of living with a screening-detected celiac disease diagnosis, illustrating the grounding of theoretically constructed subcategories reflecting feelings and attitudes, and related actions, which finally are grouped into categories reflecting experiences of living with celiac disease.

Overall, the adolescents' experiences were influenced by external structures, such as support given from significant others, CD awareness, and availability of gluten-free products. Parents played an important role as facilitators, by cooking gluten-free food at home and having contact with the school cafeteria personnel and teachers about the gluten-free diet. We noted a pattern were boys were more prone to lean on the parental support whereas girls rather took actions themselves. However, when the adolescents were at school or with friends, parents did not have ample opportunity to offer support. The crucial role of support from peers, teachers, coaches, and peers' parents was clear and often elaborated on. If present, the support was appreciated and seen both as caring and as a form of positive control. Generally, lack of knowledge and poor availability when eating out were common sources of frustration.

#### Not a big deal

These adolescents labeled CD as food intolerance, which for them was not a real disease and stressed that, in comparison to other conditions, having CD was not that hard. They considered CD in the light of its consequences, namely, that you have to stick to another diet. However, the focus group discussions gave the insight that the reaction towards living with CD as 'not a big deal' was not an immediate reaction, but rather had developed after a period of adjustment.

Girl: "You think that maybe it's going to be much worse than it is. Really it isn't that bad."

Boy: "You just think that everything will be like different, but I think nothing is really different. Except that..."

Girl: [filling in] "Yeah, just that you eat differently."

Focus group discussion

#### A fight for normalization

Adolescents associated with this category perceived living with CD as inconvenient, mainly because the gluten-free diet resulted in them being treated differently. They expressed that there was a lack of knowledge about CD in society and asked for increased awareness. Whereas girls took on an active role themselves in trying to educate friends, school personnel, and restaurant owners, boys asked for support in their efforts to be looked upon as 'normal'. They wished for the scientific community to communicate about CD in newspapers and television and to have nationwide education for school cafeteria personnel. These CD ambassadors were concerned that many people remained undiagnosed and advocated screening since they believed that more diagnosed cases would make life easier also for them.

"I, being Chairman of the student council, I push it a little. We have a gluten-free group, who meets with our school nurse and our social worker. We have also talked to the school kitchen about what we can improve with the food. Then we have baked a gluten-free selection in the cafeteria, because we had complained that we never had anything to eat."

Girl, Focus group discussion

#### A lonely struggle

This category represented the experiences of felt stigma resulting in efforts to conceal the CD. These adolescents described being exposed to various situations that made them feel vulnerable, lonely, and without sufficient support. Asking for special food was associated with a worry about being a burden to others, and to compensate, they described feeling obliged to be overly nice and helpful. Boys expressed not wanting to talk about their condition in public and some seemed not to have incorporated CD into their social identities. They themselves, and their parents, described how they for example covered their CD by claiming not to be hungry or eat the food served, even if it was not gluten-free. In contrast, girls hold on to their identity of being 'a celiac', despite the social difficulties, but expressed how the disease had put an extra burden in life by inducing feelings of being an outsider.

#### Treatment not worth the price

Even if not the dominant reaction, experiences of the CD diagnosis being too hard to handle existed. The adolescents contributing to this category were girls that, after consideration, made a decision not to adhere to the diet. Even if suffering from symptoms that were caused by CD, suffering from being different was perceived as worse.

## Discussion

This is, to our knowledge, the first qualitative study exploring daily life consequences of receiving a screening-detected CD diagnosis. We found that the diagnosis had varying impact on quality of life that related both to changes in perceived health and to the adolescents' experiences of living with CD in terms of social sacrifices.

Previous research on CD and gluten-free diet's effect on quality of life has mainly utilized quantitative methods [21-26]. Although these quantified measures may facilitate reproducibility, they do not allow for capturing the complexity of the patients' lived experiences. By using qualitative methods, our study accessed adolescents' and parents' own perspective which allowed for a holistic description of changes in perceived health as well as impact on daily life. The study was characterized by an emergent design, purposive sampling of informants, reflective field notes, and oscillation between data collection and analysis. To further increase the credibility of the study, continued peer debriefing sessions were held within the research group and an audit trail with analytical memo notes was maintained throughout the study. The integrity of the study was strengthened by the moderators of the group discussions not being involved in the health care provided to the adolescents.

Focus group discussions build on group interaction and can facilitate sharing experiences, especially when eliciting children's views [33]. However, in our study the willingness to participate in the focus group discussions may have been influenced by more positive experiences of the screening. Also, few descriptions of symptoms were communicated by the adolescents, maybe because of being reticent to share descriptions of symptoms among peers. On the other hand, the parents shared rich descriptions of their child's signs, symptoms, and well-being both before and after diagnosis and treatment. In addition, the collection of individually written narratives enabled us to capture more personal and sensitive experiences and also to explore the variation in experiences of the adolescents and their parents.

We found a large variation in perceived health before diagnosis among screening-detected CD adolescents, which has also been described by others [2,3,18,20]. Together these results confirm that not all screening-detected CD cases perceive themselves as healthy. Our findings that some had experienced health problems, and sought health care, without receiving a correct diagnosis indicate that further educational efforts to increase CD awareness are needed. The observed phenomena of retrospective recognition of symptoms in relation to a screening-detected CD diagnosis is in line with other studies [18,20], and seems to reflect both an increased understanding of symptoms and a reassurance of the benefits of having received the diagnosis.

In this study, we observed a varying impact on quality of life in terms of social sacrifices for the adolescents. Whereas some had, or were provided with, tools to successfully manage daily life, others found CD to be truly burdensome with considerable negative impact on their lives. A prominent experience was that adhering to the dietary restrictions limited daily life and caused feelings of being a burden or an outsider. Thus, adhering to the gluten-free diet related to felt stigma, as defined by Scambler and Hopkins [34]. In line with Goffman's work on stigma management [35], we found that the adolescents had adopted strategies such as withdrawing from social contacts, attempting to hide their condition, or compensating by being overly nice. These findings build on to the findings of another study on clinically diagnosed adolescents reporting on stigma experiences related to gluten-free diet [36], by indicating that mode of diagnosis probably does not affect stigma experiences.

Our results also indicated that stigma experiences may be linked to gender differences in management strategies. In general, boys described more efforts to conceal their disease and reluctance to incorporate the disease into their social identities than girls. However, those who had chosen to abandon the gluten-free diet were girls. We saw a tendency that boys asked for support in their efforts to change external structures, whereas girls took on an active role themselves. These results are in line with studies about adolescents with asthma and diabetes showing that gendered meanings of stigma influence the strategies used to cope with the disease and treatment [37].

Mass screening for CD is still questioned, although most of the World Health Organization's criteria for implementation of mass screening programs are fulfilled [11-18]. A common argument against CD mass screening is that the diagnosis and treatment would be harder to accept and manage among those experiencing no prior symptoms compared to patients with clinically-detected CD. This assumes that screening-detected CD cases do not experience symptoms, while we and others have shown that screening also captures unrecognized symptomatic cases [19,20]. Furthermore, it assumes that experiencing health improvement facilitates the acceptance of the diagnosis. However, we found that the adolescents' feelings and attitudes about living with CD did not have a direct relation to whether or not experiencing health improvement. Those with great health benefits could be the ones suffering most in terms of social consequences, and vice versa, indicating that there are many aspects, apart from perceived health benefits, influencing the adolescents' experiences. Thus, CD screening as a public health intervention needs to be evaluated by balancing intended positive outcome in terms of health benefits against unintended negative consequences in terms of social sacrifices [38,39]. Further qualitative studies on psychological and social reactions as well as attitudes and feeling towards a CD screening are needed to fully understand the implications for designing and evaluating full scale screening programs among children or adolescents. Preferably such studies should also involve other age groups and cultural settings.

## Conclusions

Screening-detected CD has varying impact on adolescents' quality of life, where their perceived change in health has to be balanced against the social sacrifices that the diagnosis may cause. This needs to be taken into account in any future suggestion for CD mass screening and in the management of these patients.

## List of abbreviations

CD: celiac disease.

## Competing interests

The authors declare that they have no competing interests.

## Authors' contributions

AR contributed to the design of the study, collected data, performed data analyses, and wrote the first and final drafts. AI and ME contributed to the design of the study and guided the data collection and analysis. KN contributed to analysis and in writing. EK assisted in the data collection. AC, LD and LH contributed to the design of the study. All authors read and revised the drafts critically and read and approved the final version.

## Pre-publication history

The pre-publication history for this paper can be accessed here:

http://www.biomedcentral.com/1471-2431/11/32/prepub
